# Monitoring the motor cortex hemodynamic response function in freely moving walking subjects: a time-domain fNIRS pilot study

**DOI:** 10.1117/1.NPh.8.1.015006

**Published:** 2021-02-22

**Authors:** Michele Lacerenza, Lorenzo Spinelli, Mauro Buttafava, Alberto Dalla Mora, Franco Zappa, Antonio Pifferi, Alberto Tosi, Bruno Cozzi, Alessandro Torricelli, Davide Contini

**Affiliations:** a Politecnico di Milano, Dipartimento di Fisica, Milano, Italy; b Istituto di Fotonica e Nanotecnologie, Consiglio Nazionale delle Ricerche, Milano, Italy; c Politecnico di Milano, Dipartimento di Elettronica, Informazione e Bioingegneria, Milano, Italy; d Università degli Studi di Padova, Dipartimento di Biomedicina Comparata e Alimentazione, Legnaro, Italy

**Keywords:** time-domain functional near-infrared spectroscopy, functional near-infrared spectroscopy, gaiting task, walking, freely moving, brain, oximetry

## Abstract

**Significance:** This study is a preliminary step toward the identification of a noninvasive and reliable tool for monitoring the presence and progress of gaiting dysfunctions.

**Aim:** We present the results of a pilot study for monitoring the motor cortex hemodynamic response function (HRF) in freely walking subjects, with time-domain functional near-infrared spectroscopy (TD fNIRS).

**Approach:** A compact and wearable single-channel TD fNIRS oximeter was employed. The lower limb motor cortex area of three healthy subjects was monitored while performing two different freely moving gaiting tasks: forward and backward walking.

**Results:** The time course of oxygenated and deoxygenated hemoglobin was measured during the different walking tasks. Brain motor cortex hemodynamic activations have been analyzed throughout an adaptive HRF fitting procedure, showing a greater involvement of motor area in the backward walking task. By comparison with the HRF obtained in a finger-tapping task performed in a still condition, we excluded any effect of motion artifacts in the gaiting tasks.

**Conclusions:** For the first time to our knowledge, the hemodynamic motor cortex response was measured by TD fNIRS during natural, freely walking exercises. The cortical response during forward and backward walking shows differences, possibly related to the diverse involvement of the motor cortex in the two types of gaiting.

## 1 Introduction

Gaiting is a natural skill, making humans able to move around in free space in upright position. Almost 65% of people over 70 years old suffer from gait disorder and this issue is often linked to neurological pathologies including Alzheimer’s, Parkinson’s, and multiple sclerosis.^1^^,^^2^ Falling is a severe consequence of walking dysfunctions, frequent in elderly population.^3^ Other than provoking injuries and physical debilitation, ambulatory difficulties lead to psychological problems such as depression or fear of falling.^4^ Gaiting disorders and related issues involve more than 1% of the total expenses for the health care system in the USA.^5^ Although this phenomenon has been widely studied,^1^ a satisfactory understanding of neuronal and cerebral hemodynamic processes occurring during a gaiting task is still lacking. Studies have been performed by functional magnetic resonance imaging (fMRI) and positron emission tomography (PET) on brain activations during imaginary walking tasks, the only way to simulate gaiting due to the severe movement limitations of these techniques. Less can be found on brain hemodynamic monitoring during real gaiting in ecological experimental conditions. Different fNIRS and fMRI studies have shown how goal-directed locomotion, such as walking on assigned steps, given path-lengths, or walking on a straight line (real or imagined), significantly affects prefrontal cortex hemodynamics, reason why many experiments have monitored these areas. However, the involvement of motor cortex areas and the comparison of goal-directed locomotion with the more natural forward walking is not clear yet.

Functional near-infrared spectroscopy (fNIRS) is a promising technique for brain monitoring in real-life settings. Being compact, non-invasive, and easy to use, it turns out to be a good candidate for measurements on freely moving subjects. A good number of studies have been performed with continuous wave (CW) fNIRS instrumentations on a wide variety of walking tasks, focusing mainly on cognitive involvement in goal-oriented gaiting and prefrontal cortex activation.^6^ However, some issues are weakening the reliability of CW-fNIRS measurements in gaiting tasks, especially high sensitivity to motion artifacts and extra-cerebral hemodynamics.^7^ Time-domain (TD) fNIRS is less sensitive to motion artifacts and can decouple information from extracerebral tissues and brain cortex more easily. Moreover, TD-fNIRS instrumentation yields absolute quantitative information on oxygenated and deoxygenated hemoglobin, known to be both needed to accurately detect functional brain activations.^8^

In this work, we used a wearable, single-channel, dual wavelengths (670 nm, 830 nm) TD-fNIRS device^9^ aiming at a reliable recording of the cortical hemodynamics from the motor area during gaiting tasks in natural conditions.

## 2 Materials and Methods

Three healthy male participants (age 30, 55, and 50 years) were included in this pilot study. All subjects cooperated voluntarily and provided written informed consent to the procedures of the study, which was approved by the Ethics Committee of Politecnico di Milano. The compact TD-NIRS device^9^^,^^10^ has been mounted on a backpack custom support to be comfortably worn by the subjects. The system was battery operated and, thanks to its lightweight (<2.5  kg) and remote control via Wi-Fi, it was possible to ensure completely free motion to all the participants.

**Fig. 1:**
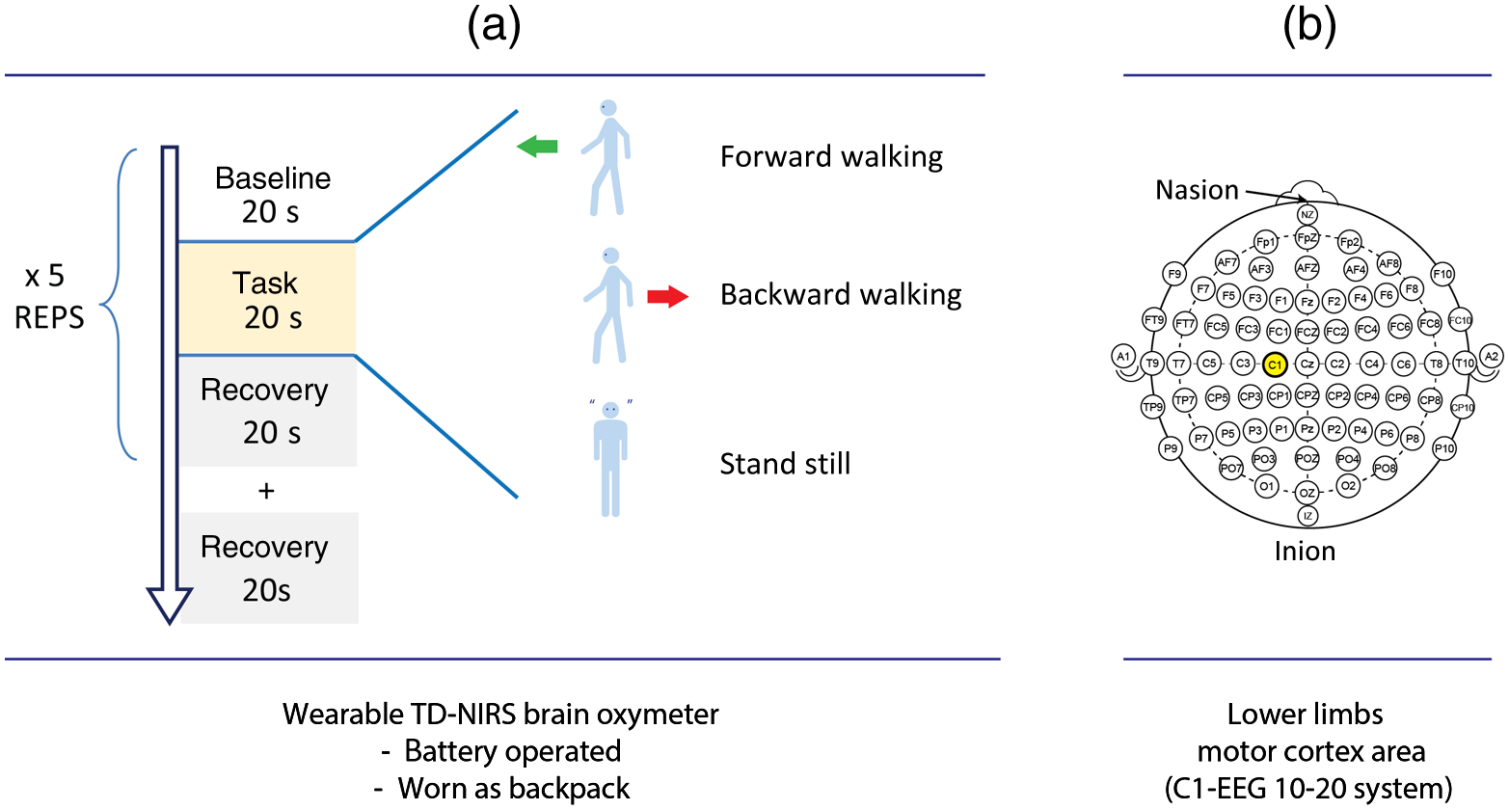
(a) Sketch showing the experimental protocol used in the study. For each task (forward walking, backward walking, and stand still), five repetitions have been performed. (b) The probe was positioned following the electroencephalography (EEG) 10/20 map system and the position was C1 in the motor cortex area.

Subjects performed three different experiments: forward walking, backward walking, and control (standstill). All measurements had the same structure consisting of five trials, lasting 60 s each: 20 s baseline with subjects standing and not moving, 20 s task with subjects walking forward/walking backward/standing still, and 20 s recovery with subjects standing still [see Fig. 1(a)].

At the end of the five repetitions, all subjects performed 20 s of extra recovery. The total experiment lasted ∼20  min, including system setup for each volunteer. For what concerns the data analysis, the 20 s recovery period in each repetition has been grouped with the 20 s baseline period of the following repetition to enlarge the total recovery time and better appreciate the full-time dynamic of the vascular response.

Probe positioning [see Fig. 1(b)] was selected based on previous works, such as Ref. 11, in which motor imagery is used to study cortical activation in forward and backward gaiting. The distance between the injection and the collection fibers on the optical probe was set to 30 mm. The region of interest was the position C1 from the EEG 10/20 system: the probe was placed with the source-detector line, parallel to the midline coronal plane, and was secured on the scalp of participants with a black elastic bandage around the head, avoiding ears and eyes coverage.

The acquisition frequency was set to 1 Hz, with 500 ms integration time per wavelength. To ensure a low intrinsic variation on the retrieved oxyhemoglobin ($O_{2}\mathrm{Hb}$) and deoxyhemoglobin (HHb) concentrations, in detail lower than 1%, the acquisition count-rate was set to about $10^{6}\;\text{photons}$ per second.^9^ Data analysis was based on the convolved photon path-length method, which showed to be a good tool to decouple deep from shallow layers’ information in TD-fNIRS.^12^ The mean values of absorption and reduced scattering coefficients in the baseline period ($\mu_{a,0}$, and ${\mu^{\prime }}_{s,0}$, respectively) are obtained by fitting the average photon distribution of time-of-flight (DTOF) with the solution of the diffusion equation for a semi-infinite homogeneous medium.^13^ Those values are then used to calculate the time-dependent mean partial path-length in extra-cerebral and brain cortex layers, assuming an equivalent superficial extra-cerebral optical thickness of 0.5 cm and dividing the DTOF curve in 10 consecutive time-gates each with 400 ps width. All gates have been used for the analysis. It was then possible to estimate the variation of the absorption coefficient ($\Delta \mu_{a}$) in the two layers for each acquisition time point t and to compute the absorption coefficient as $\mu_{a}(\lambda ,t)=\mu_{a,0}(\lambda )+\Delta \mu_{a}(\lambda ,t)$. Hemodynamic parameters can be retrieved by Beer’s law from the resulting $\mu_{a}(\lambda ,t)$, assuming that $O_{2}\mathrm{Hb}$ and HHb are the main chromophores contributing to absorption. Throughout an adaptive fitting procedure, inspired by the fNIRS study presented by Uga et al.,^14^ we retrieved the hemodynamic response function (HRF) for $O_{2}\mathrm{Hb}$ and HHb activations in both gaiting tasks. The HRF is formed by the sum of two gamma functions with opposite sign, depending on the following parameters: the amplitude (AMP) and peak position with respect to the beginning of the task ($\tau_{p}$) of the first gamma function, the ratio (β) between the amplitude of the two gamma functions, and the delay of the peak of the second gamma function ($\tau_{d}$). Following the procedures depicted in the study made by Uga et al.,^14^ β and $\tau_{d}$ parameters have been fixed to 1/6 and 10 s, respectively.

Even if in most cases a single repetition gave a sufficient contrast-to-noise ratio (CNR) on $O_{2}\mathrm{Hb}$ and HHb, the five repetitions were averaged for every exercise and every subject, to better appreciate the corresponding HRF. In the analysis, error bars show the standard deviation over the five repetitions and the black asterisks highlight the measurement points in which the variation (with respect to baseline values) is significant [interval-avlue (p)<0.05]. The p-value was evaluated using the Welch’s t-test, or unequal variances t-test, to test the hypothesis that our two populations had equal means. The expected canonical HRF shows an increase in $O_{2}\mathrm{Hb}$ and a decrease in HHb. The fitting results have been evaluated considering Pearson’s correlation coefficient (R): R<0.3 translates in weak correlation, 0.3<R<0.7 in moderate correlation and R>0.7 in high correlation.

To better understand the outcomes of the walking experiment, the results have been compared to a standard motor exercise (finger tapping) with the same timing (20 s baseline, 20 s task, 20 s recovery) previously performed and presented in another work on the same three subjects.^9^ In this test, subjects were asked to sit on a chair and the probe was placed on the C3 position of the 10/20 EEG system, probe positioning was contralateral with respect to the finger movement.

## 3 Results

**Fig. 2:**
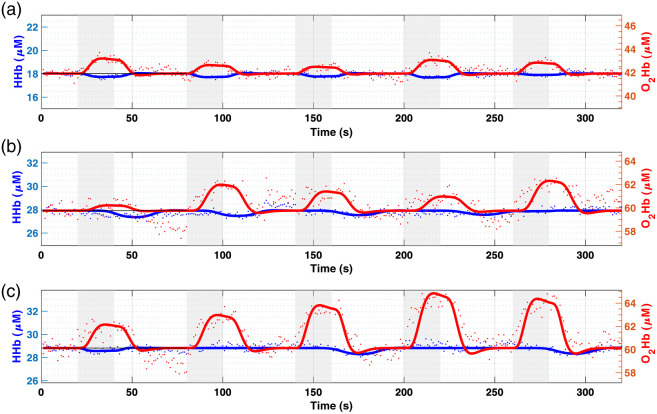
Time course of hemodynamic parameters in the three tasks for subject #3: (a) finger tapping; (b) forward walking; and (c) backward walking. Data are for one subject and five repetitions. Round dots represent raw data of $O_{2}\mathrm{Hb}$ (red) and HHb (blue) absolute concentration. Thicker lines show the fitted HRF for each repetition while gray shaded areas highlight the tasks’ periods.

As example raw data of one volunteer are shown in Fig. 2 for the three motor exercises: finger tapping, forward walking, and backward walking. For each task, graphs show absolute concentrations of $O_{2}\mathrm{Hb}$ (red) and HHb (blue) over the five repetitions. HRFs have been superimposed to the raw data. We observe a canonical HRF (increase in $O_{2}\mathrm{Hb}$ and a corresponding decrease of HHb) for each task and nearly every repetition. The first repetition of the forward walking task resulted in a lower activation intensity in all volunteers.

**Fig. 3:**
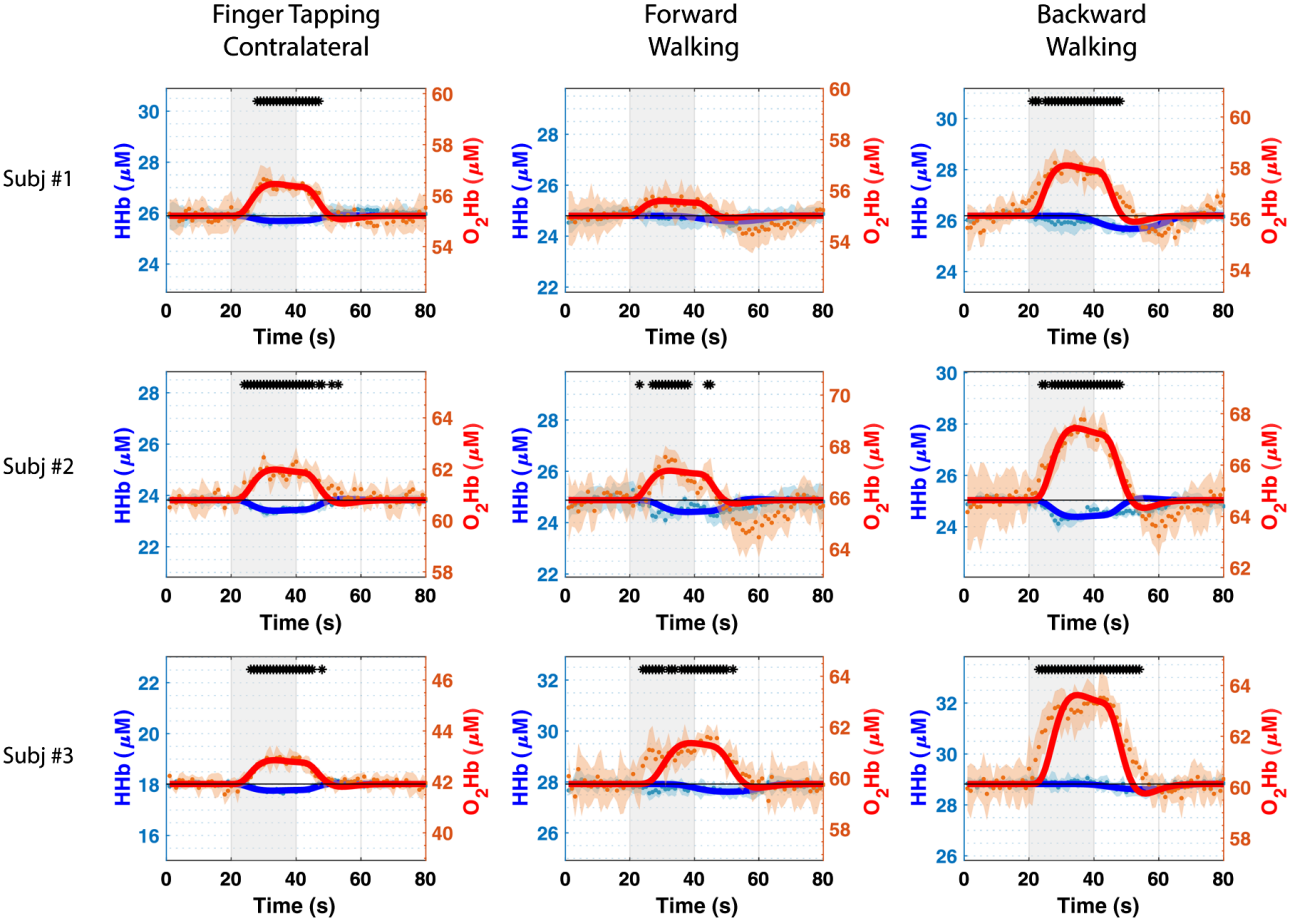
Time courses of $O_{2}\mathrm{Hb}$ (red) and HHb (blue) for the three different subjects during the three different tasks. Plots report the mean (filled dots) and standard deviation (shaded area) over the five repetitions for each time point. Black asterisks are used to highlight significant activations with respect to the baseline (p<0.05). Solid lines represent the result of the adaptive HRF fit over the averaged repetitions.

In all subjects, a substantial difference can be seen between the activation intensity related to the forward walking task and the one related to the backward walking (see Fig. 3).

In particular, the average activation intensities for $O_{2}\mathrm{Hb}$ (calculated between 20 and 50 s) of backward walking task result almost doubled compared to those of the forward walking task.

**Fig. 4:**
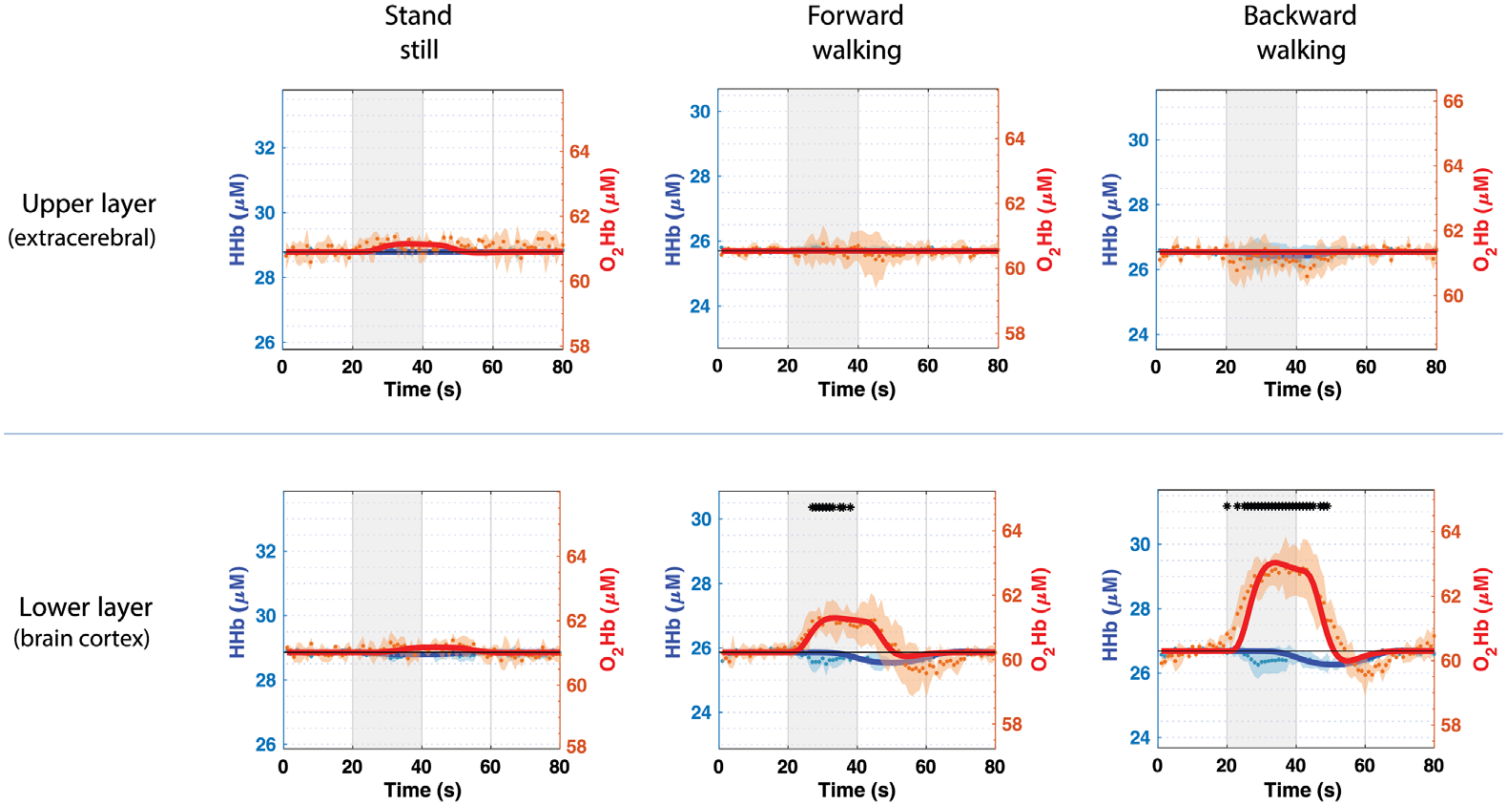
Mean over three subjects, performing five repetitions of three different tasks (see columns): standstill (control condition), forward walking, and backward walking. Round dots represent raw data of $O_{2}\mathrm{Hb}$ (red) and HHb (blue) absolute concentration in time. Thicker lines show the fitted HRF for each repetition. Gray shaded areas highlight the task time periods, and shaded error bars represent the standard deviation resulting from the five repetitions average and three different subjects. Black asterisks are used to highlight significant activations (p<0.05).

Average (standard deviation) reconstructed optical properties in baseline conditions across all subjects and experiments are: $\mu_{a}$ $670\;\mathrm{nm}=0.213\;{\mathrm{cm}}^{-1}$ (0.016), $\mu_{a}$ $830\;\mathrm{nm}=0.178\;{\mathrm{cm}}^{-1}$ (0.008), ${\mu_{s}}^{\prime }$ $670\;\mathrm{nm}=16\;{\mathrm{cm}}^{-1}$ (3), and ${\mu_{s}}^{\prime }$ $830\;\mathrm{nm}=12\;{\mathrm{cm}}^{-1}$ (1). Baseline values for subject 3 in the finger tapping protocol appears different from what was found in the other subjects. Average baseline values where although confirmed in other three finger tappings experiments performed by subject 3 the same day also after probe repositioning. We cannot exclude that such variation occurred due to a concomitant effect of physiological variation of the subject and a suboptimal probe positioning. No task-related activations have been revealed during the control condition (see Fig. 4). The analysis method exploited in this study gave us the possibility to retrieve separate information on the upper and lower layers of the probed medium. The hemodynamic variations retrieved from extra-cerebral tissue (upper layer) and cerebral tissue (lower layer) are reported in Fig. 4, in which the responses have been averaged on every subject and every repetition. Extra-cerebral tissue did not show any task-related behavior either during the motor tasks or in the control condition, whereas, as expected, we report significant average activation during forward and backward walking tasks.

From Fig. 4, it is possible to ascertain that the retrieved activation is mainly a contribution from the deeper layer, which is the cerebral cortex. On the other hand, when the subject is standing still there is no activation. In Figs. 2, 3, and 4, thicker lines show the HRF retrieved by fitting the raw data points of the hemodynamic parameters. The fitting method resulted more efficient in the case of the finger-tapping motor task, showing higher Pearson’s correlation coefficient (R=0.92 for finger tapping, R=0.78 for forward walking and R=0.80 for backward walking; averaged over the three subjects). As suggested by Fig. 3, the fitting looks less optimal in case of forward and backward walking tasks, in which the activation seems prolonged in time compared to the case of contralateral finger tapping. The HRF fit results suboptimal especially in case of the HHb behavior in the gaiting tasks. Table 1 reports the mean HRF parameters for both $O_{2}\mathrm{Hb}$ and Hb resulting from the average of the subject’s activations in the three motor exercises. From Table 1 it is possible to confirm that the amplitude (AMP) of the $O_{2}\mathrm{Hb}$ response in backward walking is significantly different (nearly double) compared to forward walking. The behavior of HHb looks generally delayed in time, showing a larger delay ($\tau_{p}$) for the HHb compared to the $O_{2}\mathrm{Hb}$.

**Table 1:** Resulting HRF parameters (AMP and $\tau_{p}$) relative to motor exercises, averaged over three healthy subjects. Variables are grouped for $O_{2}\mathrm{Hb}$ and HHb. Parameters β and $\tau_{d}$ are fixed to 1/6 and 10 s, respectively, according to Uga et al.^14^

		Contralateral finger tapping	Forward walking	Backward walking
$O_{2}\mathrm{Hb}$	AMP (STD) (μM)	1.22 (0.28)	1.11 (0.51)	2.76 (0.76)
	$\tau_{p}$ (STD) (s)	7.55 (0.36)	7.63 (3.66)	7.45 (1.47)
HHb	AMP (STD) (μM)	−0.40 (0.11)	−0.33 (0.13)	−0.49 (0.18)
	$\tau_{p}$ (STD) (s)	8.59 (2.35)	18.71 (6.90)	19.15 (9.65)

## 4 Discussion

Three male adults have been recruited to test the capability of a TD-NIRS portable instrument to study motor cortex activation in forward and backward natural gaiting tasks. The time-courses of absolute concentrations of $O_{2}\mathrm{Hb}$ and HHb have been measured in intracerebral tissue. An adaptive HRF method has been applied to preliminary characterize the responses. Results from the gaiting tasks are in good accordance with many studies performed with fMRI on motor imagery of gaiting task.^15^^–^^18^ Motor imagery is known to be a reliable method to investigate cortical activations during locomotion within fMRI scanners, and studies have confirmed that similar and identical brain areas are activated as if the movement was actually being performed. As it is clearly depicted in Ref. 11, a wider area of supplementary motor area and primary motor cortex supplementary motor area (SMA)/primary motor cortices (PM1) activates during imaginary backward gaiting together with a more intense blood oxygenation level-dependent (BOLD) signal. Our study, in which the single-channel probe was placed close to the barycentre of the activation seen in Ref. 11, indicates that even in real gaiting tasks the activation is greater (almost double) in case of backward walking. In the same work,^11^ an earlier decrease of the BOLD fMRI signal was seen in case of forward walking, resulting in a shorter activation response to the imaginary task. Comparable results were drawn from our measurements, where backward walking shows prolonged activation compared to forward gaiting for $O_{2}\mathrm{Hb}$ concentration. Godde and Voelcker-Rehage^11^ also showed that the barycentre of both imaginary gaiting tasks is ∼2.5-cm deep, which is within our TD-NIRS system capabilities. Our results are in accordance with other CW-fNIRS studies, even if performed in less natural and ecological environments.^19^

CW-fNIRS relies on measuring light attenuation and this is prone to artifacts due to poor or loose coupling of the sensors with the scalp. Indeed a poor or loose coupling can change the measured attenuation in a way that might not be distinguishable from a change induced by, e.g., a functional activation. Noticeably, motion artifacts can be identified on the basis of the temporal evolution of the NIRS signal, being the HRF rather slow, any fast or abrut change can be attributed to motion artifacts.^7^ On the contrary, a change due to poor or loose coupling of the probe might be well tolerated by TD NIRS if it simply determines a change in the collected photon count rate. This change will affect in a similar way both early and late photons and by a proper regression procedure it can be compensated. On the other hand, if the motion artifact is related to stray laser light not passing trough the tissue, it will introduce a distortion in the DTOF (e.g., early peak or time broadening) and it can be easily identified.

A detailed analysis of the neural circuitry and pathways responsible for the greater activation detected in the motor cortex during backward walking is outside the scope of the present article. However, as a preliminary hypothesis, we emphasize that forward walking relies on visual cues and involves the corticospinal tract^20^ with possible involvement of the pattern generators in the brainstem. Backward walking, on the other hand, lacks visual directions, requires increased consideration of proprioceptive signals, direct pyramidal control of muscle coordination, and possibly suppression of extra-pyramidal activities and consequent motor schemes.^21^ The net result of these latter actions may convey in an increased direct activity of the motor cortex.

Nearly all subjects showed significant cerebral activation in every motor exercise. Only in the case of subject 1, the average activation resulting from the forward walking protocol was not strong enough to be considered significant. This could be due to the easiness of the proposed exercise that could be performed mechanically and almost instinctively by the subject.

Many studies have been performed on the correlation between gaiting speed and motor cortex activation intensities^22^^,^^23^ showing greater involvement of SMA and SM1 areas in case of shorter stride-time.^19^ It was therefore our concern to keep the walking speed as constant as possible to prevent additional variations related to different gaiting speeds in both tasks: forward and backward gaiting. The preparatory phase was assessed to be determinant in prefrontal and SMA cortex hemodynamic before and during the gaiting task, showing increased activations in case of presence of preparatory instructions.^24^ We, therefore, decided to have an identical preparatory section in all the three protocols regarding forward walking, backward walking, and control. Preparatory time consisted of 3 s, included in the baseline section, in which the supervisor of the experiment counted backward from 3 to 1 and gave the start command.

The main limitation of our study is the number of measured subjects, nevertheless, this issue has been partially solved by the high CNR retrieved from the measurements, the high number of task blocks taken into account (45 blocks, 15 per subject), and the presence of a control condition. A further limitation can be addressed to the single-channel probe. Even if the target area was specifically the lower limb motor cortex (the region around C1/C2 EEG 10-20 system), one single channel limits the probability of positioning the probe in the right spot. In particular, for the forward gaiting, in which a smaller brain region is expected to activate, it cannot be excluded that the location of the probe was sub-optimal, causing a less efficient acquisition of the hemodynamic variations. In future studies, more channels would ensure a more robust positioning of the probe, and a wider cohort of subjects would lead to a complete characterization of the HRF for backward and forward gaiting.

Most of the fNIRS studies involving gaiting tasks have been designed with the use of extra mechanical equipment, such as treadmill,^25^ preventing the ecological environment that offers natural waking. Moreover, to the best of our knowledge, no studies have yet applied TD-fNIRS brain monitoring to freely walking subjects, probably due to lack of compact TD-fNIRS devices. Nevertheless, such a technique could give great advantages to the removal of moving artifacts. Compact, accurate, and reliable TD-fNIRS instrumentation could eventually lead to the possibility of diagnosing gaiting pathologies or related neurological issues, with less economic impact on the health system. In the future, diagnostic-oriented studies can eventually focus on the activation variability that has been found in older adults, compared to younger ones, for what concerns complex gaiting tasks. In fact, elderly individuals show less selective recruitment of brain areas than younger.^26^ Stronger activations of sparse brain areas can suggest a lower capability to appropriately address specific neuronal mechanisms.^27^ In such a population, and also in neurological patients, it can be hypothesized that the difference in the activation between the two studied tasks is lowered due to the recruitment of different zones limiting the implication of the M1 area. A quantitative test, such as the one proposed in this study, can be a possible diagnostic tool to evaluate the entity and evolution of those neurological pathologies that directly affect stable gaiting.

## 5 Conclusion

In this work, we used a wearable, single-channel TD-fNIRS device, developed at Politecnico di Milano,^9^^,^^10^ for a pilot study on volunteers during forward and backward walking. The system was mounted in a backpack configuration and proved to be rugged and reliable, allowing the estimation of cortical hemodynamics from the motor area during gaiting tasks in natural conditions. The cortical response was fitted to an HRF model, and different parameters were obtained during forward and backward walking, possibly related to the diverse involvement of the motor cortex in gaiting. Future work will be focused on increasing the number of channels for mapping the motor cortex and on recording from healthy and pathological subjects.

## Code, Data, and Materials Availability

The data that support the findings of this study are available from the corresponding author, D. Contini, upon reasonable request.
